# Deciphering *TCOF1* mutations in Chinese Treacher Collins syndrome patients: insights into pathogenesis and transcriptional disruption

**DOI:** 10.1186/s13023-024-03508-z

**Published:** 2025-02-07

**Authors:** Zhuoyuan Jiang, Ke Mao, Bingqing Wang, Hao Zhu, Jiqiang Liu, Ruirui Lang, Baichuan Xiao, Hailin Shan, Qi Chen, Ying Li, Shouqin Zhao, Qingguo Zhang, Huisheng Liu, Yong-Biao Zhang

**Affiliations:** 1 School of Engineering Medicine, Beihang University, Beijing, China; 2 Department of Ear Reconstruction, Plastic Surgery Hospital, Chinese Academy of Medical Sciences, Beijing, China; 3 Department of Otolaryngology Head and Neck Surgery, Beijing Tongren Hospital, Capital Medical University, Beijing, China; 4 Bioland Laboratory, Guangzhou Regenerative Medicine and Health Guangdong Laboratory, Guangzhou, China; 5 Key Laboratory of Big Data-Based Precision Medicine (Beihang University), Ministry of Industry and Information Technology, Beijing, China; 6 Guangzhou National Laboratory, Guangzhou, 510320 China

**Keywords:** Treacher Collins syndrome, *TCOF1*, Frameshift, Pathogenic, Intrinsically disordered protein

## Abstract

**Background:**

Treacher Collins syndrome (TCS, MIM #154500), a severe congenital disorder, predominantly involves dysplasia of craniofacial bones and is characterized by features such as downslanting palpebral fissures, lower eyelid colobomas, microtia, and other craniofacial anomalies. Despite its clinical importance, the underlying pathogenic mutations in TCS remain largely uncharacterized, representing a critical knowledge gap for researchers in the field.

**Results:**

To address this, we performed mutation screening on a familial TCS case (trio) and 11 sporadic cases from a Chinese population. We identified 11 mutations predominantly localized to the central repeat domain (CRD) and the C-terminal domain (CTD, including the nuclear localization sequence) of TCOF1. The de novo frameshift mutation identified in the trio led to TCOF1 truncation, disrupting the central repeat domain crucial for binding transcriptional factors. Immunoprecipitation assays revealed that this pathogenic mutation attenuates the interaction between TCOF1 and transcription-related proteins, such as Pol II. Furthermore, cellular luciferase assays demonstrated that the mutation compromises the nuclear localization capability of TCOF1.

**Conclusions:**

Our findings establish *TCOF1* as the primary pathogenic gene in this Chinese TCS cohort, with mutations predominantly in the CRD and CTD, thereby expanding the known mutation spectrum of TCS and informing its prevention strategies.

**Supplementary Information:**

The online version contains supplementary material available at 10.1186/s13023-024-03508-z.

## Background

Treacher Collins syndrome (TCS) represents a rare congenital anomaly, primarily manifesting as craniofacial malformations with an incidence of about 1 in 50,000 live births [1, 2]. TCS exhibits a wide phenotypic spectrum, encompassing anomalies such as facial bone aplasia, eyelid coloboma, micrognathia, cleft lip and palate, auricular deformities, and atresia of the external auditory canal. This syndrome significantly impacts patients both cosmetically and functionally, leading to diminished quality of life, including mental health challenges [2, 3]. Furthermore, the need for interventions such as hearing restoration and reconstructive surgery in TCS patients imposes a substantial burden on healthcare systems [4].

The etiology of TCS is linked to the dysmorphogenesis of the first and second branchial arches, embryonic structures rich in cranial neural crest cells (CNCCs). CNCCs play a critical role in craniofacial tissue formation [5], and it was hypothesized that mutations affecting CNCCs development contribute to the pathogenesis of TCS [6]. Initially, chromosomal abnormalities were identified as significant genetic risk factors in the manifestation of TCS [7]. The gene *TCOF1* was first identified as a pathogenic factor in TCS by Dixon et al*.* [8], followed by the discovery of *POLR1C* and *POLR1D* as additional causative genes [9]. Mutations in these genes are associated with disturbances in neural crest cells (NCCs), a key process implicated in the development of TCS.

In this study, we analyzed 11 Chinese individuals with sporadic TCS and one trio family, utilizing whole-exome sequencing and Sanger sequencing to delineate the mutation spectrum characteristic of TCS in this population. We identified 10 mutations within the CRD and CTD (with nuclear localization signal (NLS)) of TCOF1 and functional experiments demonstrated that a specific de novo frameshift mutation impairs both the binding capability and cellular localization of TCOF1. Our findings offer valuable insights for the diagnosis and potential therapeutic strategies for TCS, particularly within the context of the Chinese population.

## Methods

### Ethics statement

The study was approved by the Ethics Committees of the Plastic Surgery Hospital of the Peking Union Medical College, Beijing Tongren Hospital of Capital Medical University, and School of Biological Sciences and Medical Engineering, Beihang University. Informed consent was obtained from the patients' parents or guardians.

### Samples

**Fig. 1:**
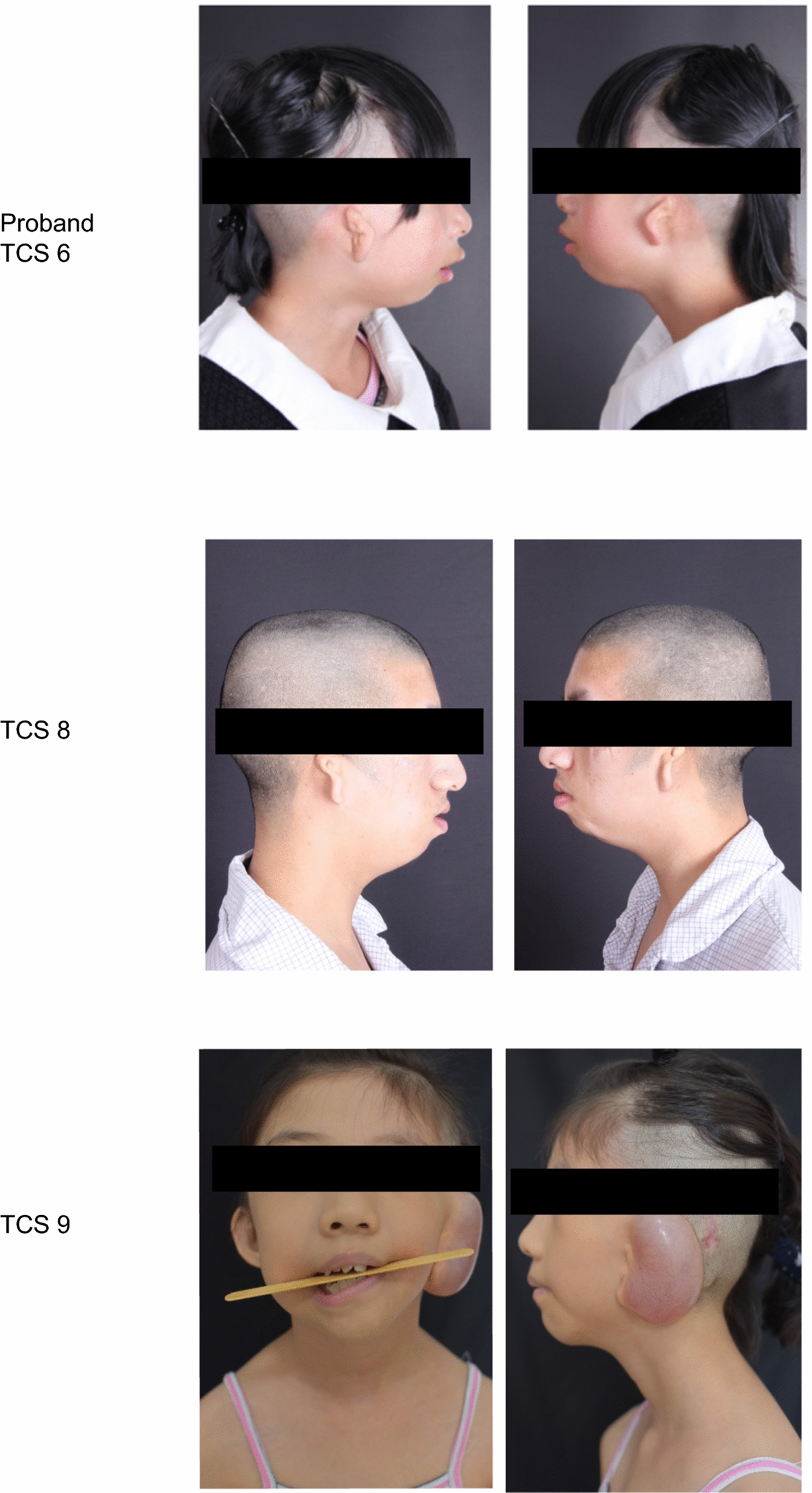
Clinical facial representations of selected patients. This figure displays the facial photographs of three individuals diagnosed with Treacher Collins syndrome (TCS), illustrating typical phenotypic features associated with the condition

We recruited 12 patients diagnosed with Treacher Collins syndrome (TCS), including a trio family, from the Plastic Surgery Hospital of Peking Union Medical College and Beijing Tongren Hospital, Capital Medical University. Comprehensive clinical data, photographic records, and auditory assessments were systematically collected for all TCS patients. Within the trio, a 10-year-old girl (designated as TCS6, as shown in Fig. 1 and Table 1), was diagnosed with TCS, while both parents exhibited no symptomatic evidence of the syndrome. Peripheral blood samples (3–5 ml) were obtained from each patient using EDTA as an anticoagulant. Subsequently, DNA was extracted using a Tiangen Biotech DNA extraction kit (Beijing, China), following the manufacturer’s protocol.

**Table 1:** Pathogenic mutations identified in the *TCOF1* gene and clinical manifestations in TCS patients

Patient	Phenotype	Location	cDNA (NM_001371623)	Protein	Domain	ESM1b	CADD
TCS 1	MD; DP; M	exon7	c.732G > C	p.(K244N)	CRD	− 8.1	23.4
TCS 2	MD: M	exon7	c.853G > C	p.(A285P)	CRD	− 6.44	22.4
TCS 3	MD; DP; M	exon9	c.1154G > C	p.(G385A)	CRD	− 8.51	20.9
TCS 5	MD; DP; M	exon10	c.1445C > T	p.(S482L)	CRD	− 9.71	26.1
TCS 6	MD; DP; M	exon11	c.1559_1571del	p.(A521Hfs*71)	CRD	–	–
TCS 8	MD; DP; M	exon12	c.1716dupG	p.(N574Efs*29)	CRD	–	–
TCS 9	MD; DP; M	exon17	c.2771dupC c.2774_2775insACACCTCCTG	p.(Q925Pfs*7) p.(A926Hfs*9)	–	–	–
TCS 10	MD; DP; M	exon25	c.4386delA	p.(K1464Rfs*112)	CTD (NLS)	–	–
TCS 11	MD; DP; M	exon25	c.4432_4434del	p.(K1483del)	CTD (NLS)	–	–
TCS 12	MD; DP; M	exon26	c.4465G > A	p.(V1489I)	CTD (NLS)	− 5.24	22.5

TCS, Treacher Collins syndrome; MD, maxillofacial dysplasia; DP, downslanting palpebral; M, microtia; CRD, central repeat domain; CTD, C-terminal domain; NLS, nuclear localization signal; CADD, combined annotation dependent depletion

### Whole-exome sequencing

The SureSelect Human All Exon 70 Mb kit (Agilent Technologies) was used for whole-exome capture. An Illumina HiSeq X10 sequencer (Illumina) was used to conduct paired-end sequencing, and the read length of each sample was 150-bp. All paired reads were mapped to the human reference genome (hg19) using Burrows-Wheeler Aligner (BWA, version 0.7.15). Picard software (version 1.92) was used to remove PCR duplicates. GATK (version 4.0) was used to detect single-nucleotide variants and small indels. For the trio, three inherited patterns were adopted for filtering variants: recessive, de novo, and compound heterozygous. Next, additional filtration was based on published mutations from the Database of Single Nucleotide Polymorphism (dbSNP v150, https://www.ncbi.nlm.nih.gov/snp/) with a cutoff of minor allele frequency > 0.001. ANNOVAR (http://annovar.openbioinformatics.org/) was used for genetic variant annotation, and deleterious variants (PolyPhen score ≥ 0.9 or SIFT score < 0.05, or CADD > 15, or ESM1b score < − 5) were kept as candidates. We defined mutations that were not found in the Genome Aggregation Database (gnomAD) and 1000 Genome (1000G) as novel.

### Sanger sequencing

We confirmed the identified mutations using Sanger sequencing for validation. *TCOF1* gene segments were amplified using polymerase chain reaction (PCR) under optimized conditions. Specific primers for PCR were designed utilizing Primer Premier 5 software (version 0.4.0). Sanger sequencing products were analyzed using an ABI 3730 genetic analyzer. Subsequent analysis of the raw sequence data was conducted using Chromas software (version 1.0.0.1).

### Functional prediction

The secondary structures of the wild-type and mutant proteins were predicted using PSIPRED (version 4.0; http://bioinf.cs.ucl.ac.uk/psipred/). Intrinsically disordered protein was predicted using IUPred2A (https://iupred2a.elte.hu/). Regions of disorder and disordered binding were determined using IUPred2 and ANCHOR2, respectively. Amino acids with IUPred2 scores larger than 0.5 indicated a disordered region. The 3D structure of TCOF1 was predicted using AlphaFold2 (https://alphafold.ebi.ac.uk/).

### hTCOF1 WT and mutant protein constructs

Human TCOF1 expression plasmids were constructed using the TCOF1 ORF (NM_001371623.1). C-terminal V5–tagged human TCOF1 over-expression plasmid (pN1-hTCOF1-WT-V5) and C-terminal V5–tagged human TCOF1 mutation plasmid (pN1-hTCOF1-mut-V5) were constructed by ligating the WT and mutation fragments into pEGFP-N1 vector at the EcoR I and Not I restriction sites. All procedures were based on standard cloning methods.

### Cell culture and transfection

All cells were cultured in Dulbecco’s modified essential medium (DMEM) supplemented with 10% (v/v) fetal bovine serum (FBS) (Gibco), penicillin/streptomycin (Gibco) at 37 °C in 5% CO_2_ and maintained under antibiotic selection for 293T cells. PolyJet transfection reagent (SignaGen Laboratories, http://signagen.com) was performed on 293T cells and Hela in pull-down and immunofluorescence experiments according to the manufacturer’s instructions and then incubated at 37 °C for 36 h. Subsequent experiments were performed 36 h post‑transfection.

### Pull-down

At 36 h after transfection, the cells were harvested and total protein was extracted from the cell lysate with IP lysis buffer (Thermo Scientific, 87,787) with protease inhibitor cocktail (MilliporeSigma, P8340) and phosphatase inhibitor (MilliporeSigma P0044). The protein lysates were incubated with anti–V5-tag mAb Magnetic Beads (MBL International Corp., M167-11) at room temperature for 3 h. Bound proteins after washing with IP lysis buffer were resuspended with 1 × loading buffer diluted by lysate and boiled for 3 min at 97 °C.

### Western blot analysis

Human TCOF1 lysates were clarified by centrifugation at 16,000 × g for 10 min at 4 °C. Protein concentration was measured with the BCA protein kit (Thermo Fisher Scientific). Lysates were resolved on 8 to 10% SDS–polyacrylamide gel electrophoresis (SDS-PAGE) gels, depending on molecular weight of proteins assessed and immunoblotted. NE-PER nuclear and cytoplasmic extraction kit (Thermo Fisher Scientific) was used to isolate nuclear and cytoplasmic fractions according to manufacturer’s instructions. Each lane contained 10–15 μg of total protein. Band density for the protein of interest was normalized to either GAPDH or α-tubulin. The following antibodies were used in this study: V5 tag (Abcam, catalog ab15828), RNA polymerase I RPA 194 (Sabta cruz, catalog 48,385), RNA polymerase II (Abcam, catalog ab5095), HDAC1 (Abcam, catalog ab109411), GAPDH (Cell Signaling Technology, catalog A2188), and α-Tubulin (Proteintech, catalog 66,031–1-IG).

#### Fluorescence and confocal microscopy

For staining 293T cells were seeded on cover-glasses, and grown at 37˚C. After transfection. Cells were fixed for 10 min using 4% para-formaldehyde. Cells were washed (3 times with PBS), and permeabilization was performed using Triton X-100, at 0.1% dilution in PBS for 3 min. Samples were blocked using BSA at 5% (w/v) dilution in PBS for 1 h at room temperature. Primary antibodies were diluted in 1% (w/v) BSA and incubated at 4 °C overnight. Coverslips were washed (3 times with PBS) and incubated with secondary antibodies (Alexa Fluor secondary antibodies, abcam), then diluted in 1% (w/v) BSA. Then cells were staining with DAPI (thermofisher, catalog P36931). Confocal imaging was performed using commercial super-resolution microscope (HIS-SIM). Immunofluorescence experiments were performed at least twice in independently.

## Results

### Patients

The study cohort comprised 5 female and 7 male patients diagnosed with TCS. Our cohort included one familial case (TCS6), while the remaining patients presented with sporadic instances of the syndrome. Clinical examination revealed that maxillofacial dysplasia was the predominant feature, present in all 12 patients. Additionally, downslanting palpebral fissures and microtia were observed in 10 of these patients (Table 1). In detail, the clinical features of Patient 6, the proband, included: coloboma of the lower eyelids, manifesting as V-shaped depressions and ptosis; short, downward-slanting palpebral fissures; an inferiorly displaced lateral canthus; zygomatic, maxillary, and mandibular dysplasia with microgenia; no apparent occlusal plane inclination, noticeable chin retraction, uneven dentition, and limited mouth opening to the width of two fingers; bilateral auricular abnormalities, characterized by peanut-shaped residual ears; external auditory canal atresia; symmetrical earlobes without clefts, preauricular fistulas, or appendages; and bilateral hearing impairment (Fig. 1).

### Pathogenic mutation identification

**Fig. 2:**
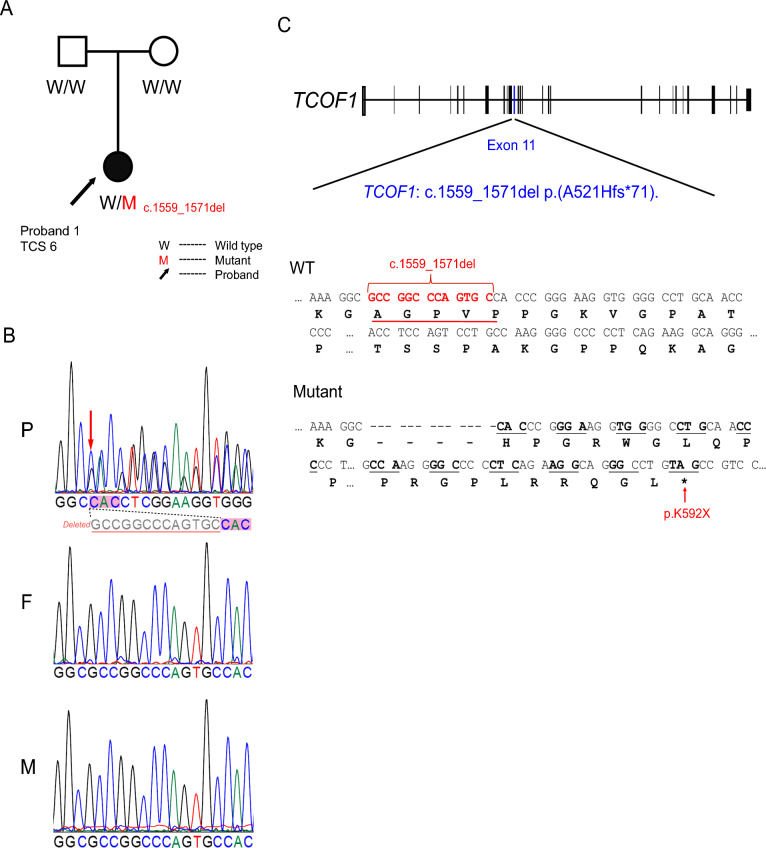
Identification and characterization of a de novo frameshift mutation in a Chinese TCS family. **A** Trio family tree: this panel illustrates the family tree of the Chinese TCS family carrying the *TCOF1* c.1559_1571del p.(A521Hfs*71) mutation. Squares represent male family members, circles represent females, and the individual with TCS (proband, TCS6) is indicated with black shading. **B** Sanger sequencing results: this panel displays the Sanger sequencing data. ‘P’ denotes the proband, ‘F’ the father, and ‘M’ the mother. The grey highlight marks the deleted nucleotide sequences, confirming the absence of this mutation in the parents. **C** Impact of mutation on TCOF1 protein: this panel shows how the mutation leads to a frameshift in the amino acid sequence, resulting in a premature stop codon (p.K592X) and subsequent truncation of the TCOF1 protein. WT, wild-type)

Whole-exome sequencing was conducted on 14 samples, comprising 11 sporadic TCS patients and one trio family. Following variant filtering based on our established criteria, we identified 11 deleterious mutations (CADD score > 15 and ESM1b Score < −5) within the *TCOF1* gene, the key pathogenic locus for TCS, across 10 patients (Table 1). The mutations comprised five frameshift, one non-frameshift deletion, and five missense mutations. Except for one mutation (c.732G > C) reported in the gnomAD database, all identified mutations were novel. Specifically, in patient TCS6, a de novo mutation, *TCOF1* NM_001371623:c.1559_1571del p.(A521Hfs*71), was identified and confirmed by Sanger sequencing. (Fig. 2A, B). This mutation, situated in the 11th exon of *TCOF1*, involved a 13 bp deletion in the coding sequence, leading to the introduction of a premature termination codon (p.K592X) and resultant truncated TCOF1 protein (Fig. 2C).

### In silico analysis of the mutant protein

**Fig. 3:**
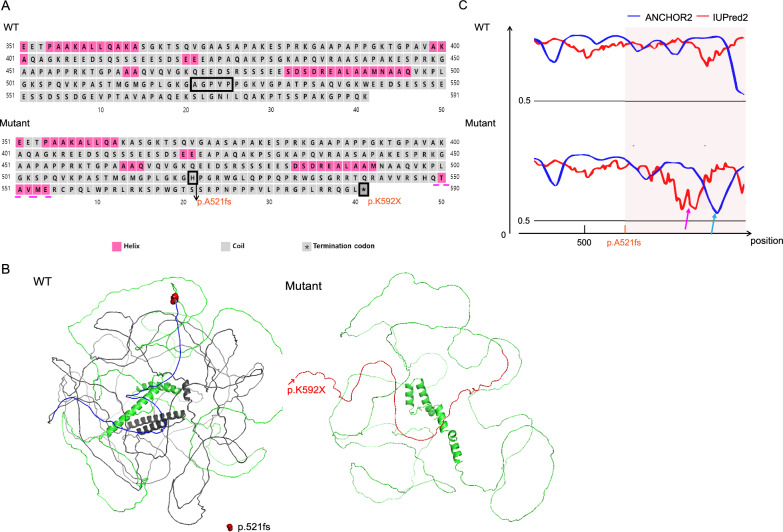
Molecular modeling predictions of TCOF1 structure and intrinsically disordered regions (IDRs). **A** Amino acid sequence alterations at mutation sites: the sequences at the mutation sites are highlighted with a dark frame. The frameshift mutation TCOF1: p.(A521Hfs*71) generates a new α-helix (indicated by a pink dotted line) and introduces a premature termination codon (p.K592X), leading to protein truncation. **B** IDR predictions for wild type and mutant TCOF1: this panel compares the predicted IDRs of the wild-type and mutant TCOF1. The mutation potentially alters the disorder degree in the TCOF1 protein, as suggested by a lower IUPred2 score (indicated by a pink arrow). The decline in the ANCHOR2 score (blue arrow) implies that the mutation might disrupt TCOF1 IDR’s binding affinity to other proteins. **C** 3D structural prediction of TCOF1: the three-dimensional structure of TCOF1 (UniProt: Q13428, AF-Q13428-F1) as predicted by AlphaFold2. The location of the frameshift mutation is marked in red. WT, wild type; IDRs, intrinsically disordered regions

IUPred2A predictions indicate that approximately 73% of the amino acids in TCOF1 are situated within intrinsically disordered regions (IDRs) (Figure S1). The TCOF1 mutation p.(A521Hfs*71) results in a truncation and the formation of an α-helix between the mutation site and the premature stop codon (p.K592X), potentially altering the properties of the disordered sequence (Fig. 3A). Additionally, this mutation is located in the central repeat domain (CRD) of TCOF1. The resulting termination codon disrupts the CRD, leading to the loss of the C-terminus. This suggests a compromised ability of TCOF1 to interact with other transcriptional regulators [2] (Fig. 3B). Moreover, the formation of this new α-helix may modify the conformation of TCOF1's IDRs, as reflected by a decreased IUPred2 score. A concurrent reduction in the ANCHOR2 score suggests that the mutation could impair the binding affinity of TCOF1's IDRs to other proteins (Fig. 2C).

### TCOF1 truncating mutations impair nuclear localization and transcription complex formation

**Fig. 4:**
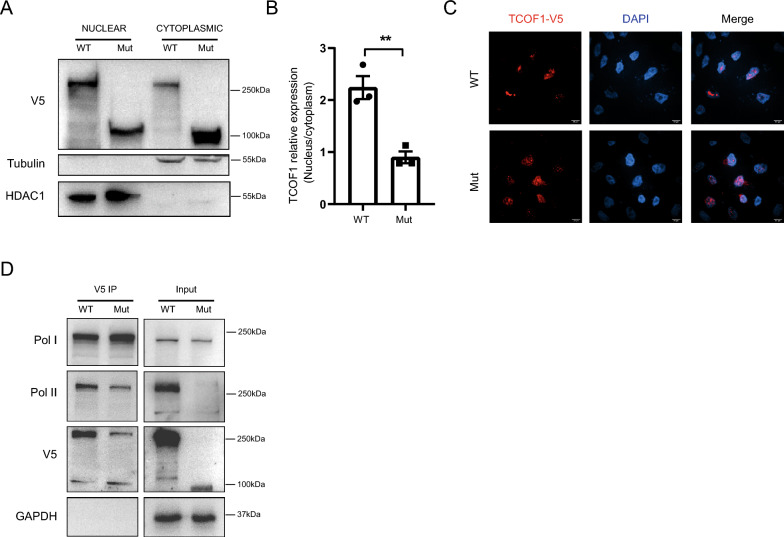
Impact of TCOF1 mutation on nuclear localization and protein–protein interactions. **A** Nucleo-cytoplasmic fractionation analysis: immunoblotting results post nucleo-cytoplasmic fractionation of HEK293T cells expressing either wild type (WT) or mutant (Mut) TCOF1. **B** Quantitative analysis of TCOF1 localization: this panel presents the quantification of TCOF1-V5 localization in the nucleus and cytoplasm from the data in (**A**). The results are expressed as means ± SEMs from three independent experiments. Statistical significance is indicated (*P < 0.05; **P < 0.01; ***P < 0.001, using One-way ANOVA). **C** Fluorescence imaging of TCOF1 localization: representative fluorescence microscopy images showing the localization of WT and Mut TCOF1-EGFP in transfected HEK293T cells. Nuclei are counterstained with DAPI. **D** Immunoprecipitation and immunoblotting analysis: Lysates from 293T cells transfected with either WT or Mut TCOF1-V5 were subjected to immunoprecipitation using an anti-V5 antibody, followed by immunoblotting with anti-Pol I/II and anti-V5 antibodies

TCOF1 plays a pivotal role in pre-rRNA transcription and processing, as well as in the spatial organization of the nucleolus. TCOF1 possesses two nuclear localization signals: one located at the N-terminus and another at the C-terminus [10]. The mutation NM_001371623:c.1559_1571del p.(A521Hfs*71) results in the truncation of TCOF1, leading to the loss of both the CRD and the CTD with NLS, potentially impairing TCOF1's ability to translocate into the nucleus. To investigate this hypothesis, we isolated nuclear and cytoplasmic fractions from 293T cells transfected with either wild-type (WT) or mutant TCOF1 plasmids. Contrasting with the WT, the truncated TCOF1 predominantly localized in the cytoplasm, with minimal nuclear presence observed (Fig. 4A, B). Furthermore, fluorescence microscopy demonstrated the inability of the truncated protein to enter the nucleus in HeLa cells (Fig. 4C).

Previous studies have demonstrated that TCOF1 interacts with RNA Polymerase I (Pol I) through its CRD and with RNA Polymerase II (Pol II) via its CTD [10, 11]. We hypothesized that the mutated TCOF1 might disrupt these interactions. To test this, we expressed both wild-type (WT) and truncated variants of TCOF1 in 293T cells to assess how these interactions are affected. The pull-down assay revealed that both WT and truncated TCOF1 successfully precipitated Pol I (Fig. 4D, upper panel). This indicates that despite the disruption of the CRD, the remaining portion in the truncated protein retained the ability to bind to Pol I, aligning with findings from previous studies [10]. Conversely, the deletion mutant of TCOF1 demonstrated an incomplete immunoprecipitation of Pol II, suggesting that the mutation adversely affects this interaction (Fig. 4D, the second panel).

## Discussion

**Fig. 5:**
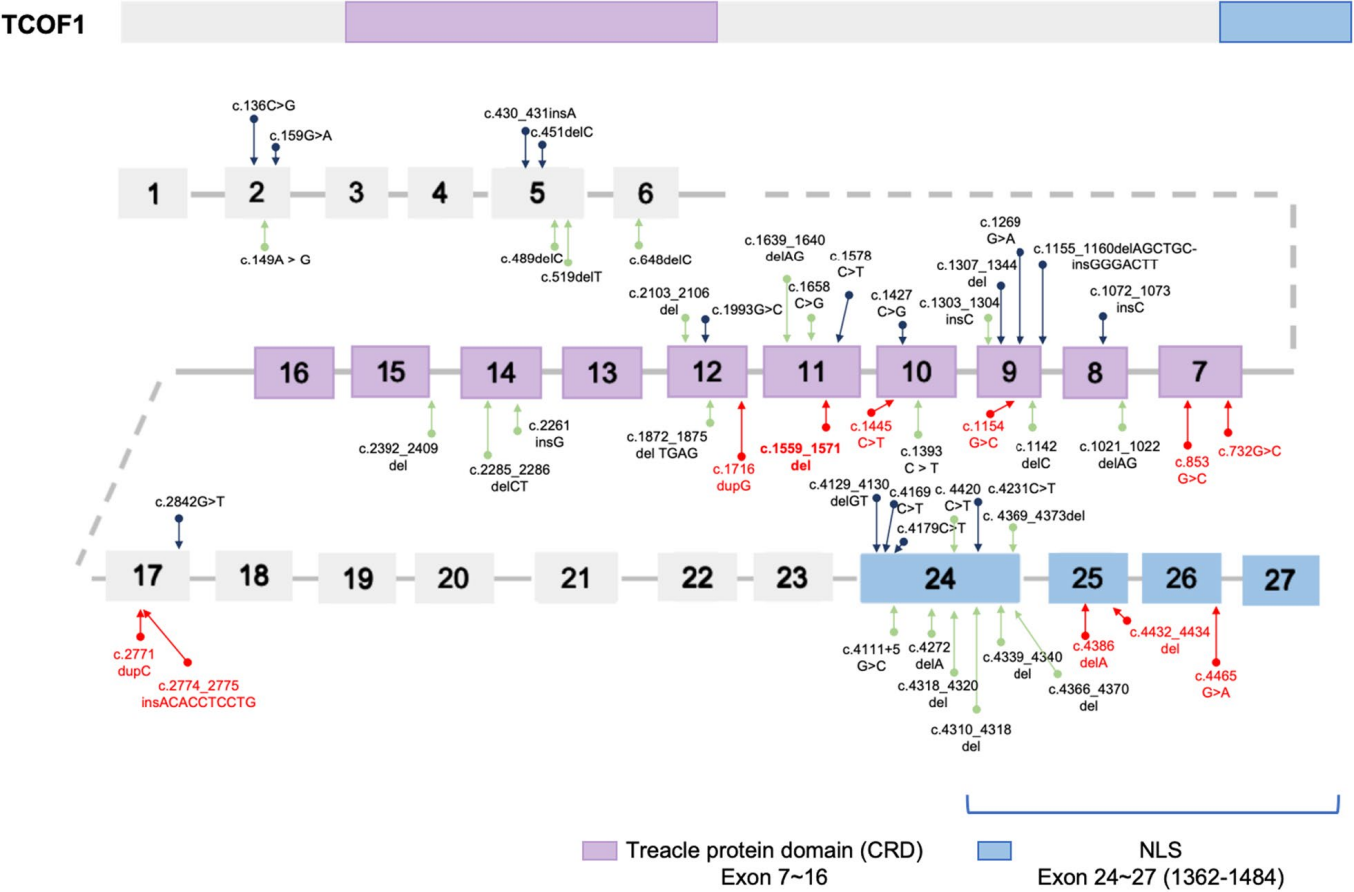
Distribution of mutations in the *TCOF1* gene among TCS patients. This schematic illustrates the causative mutations identified in the *TCOF1* gene, with exons represented proportionally by boxes. Introns and mutations in non-coding regions are omitted for clarity. The mutations are indicated by arrows, with those found in Chinese patients highlighted in green. The mutation identified in our study is specifically marked in red. Notably, mutations are predominantly located in the central repeat domain (CRD) and the C-terminal nuclear localization region

*TCOF1* has been identified as the principal pathogenic gene in approximately 78–93% of TCS cases [12]. To date, over 250 mutations in TCOF1 have been documented, yet the causative pathogenic variants in about 11% of TCS cases remain unidentified [13–15]. Gene analysis in Chinese TCS patients has revealed 19 distinct causative mutations (Fig. 5, green arrows) [13]. In both these reported cases and the 10 patients from our study, we observed that mutations predominantly occur in the Central Repeat Domain (CRD) and the C-terminal Nuclear Localization Sequence (CTD with NLS) (Fig. 5). Our findings align with published literature, which suggests that exons 10, 15, 16, 23, and 24 of the *TCOF1* gene are mutation hotspots. This pattern is also consistent in our cohort of Chinese TCS patients.

**Fig. 6:**
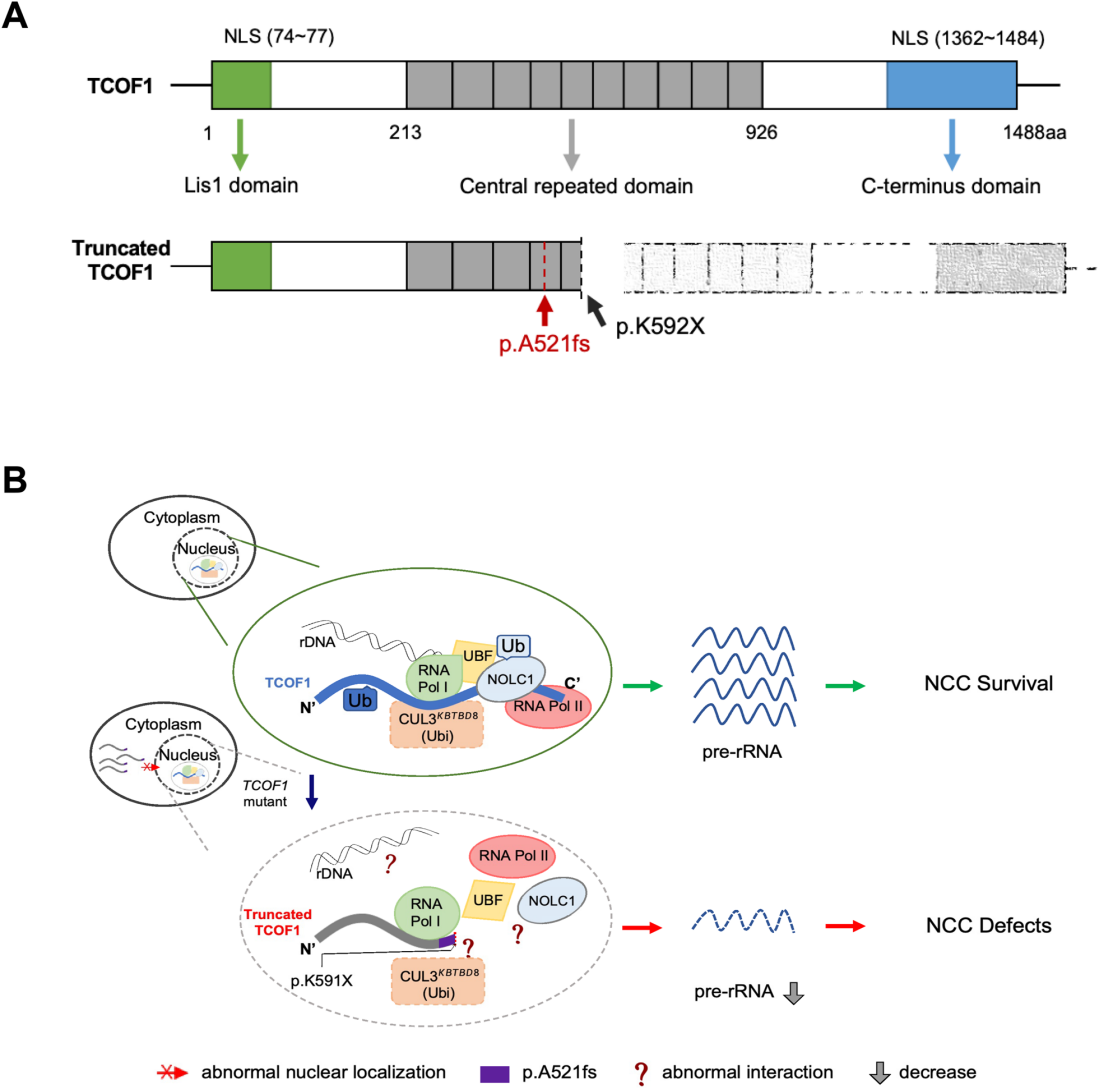
Proposed model illustrating TCOF1 domains and the etiology of mutant TCOF1. **A** Domain structure of TCOF1: this ideogram depicts the specific domains within TCOF1, including the Lis1 homology domain, the central repeat domain, and the C-terminus domain. The frameshift mutation TCOF1: p.(A521Hfs*71) results in the loss of both the central repeat domain and the C-terminus domain. **B** Etiological implications of TCOF1 mutations: the schematic represents the role of CUL^3KBTBD8^ in inducing the ubiquitin-dependent formation of the TCOF1–NOLC1 complex, which is pivotal for connecting RNA polymerase I/II to transcription-related enzymes involved in ribosomal processing and modification. Mutations in TCOF1, such as the one studied here, may disrupt this complex formation, leading to a reduction in pre-rRNA synthesis and consequent defects in NCCs

*TCOF1* encodes the nucleolar protein Treacle, comprising three distinct domains: a unique amino Lis1-homology domain, the C-terminus domain, and a characteristic Central Repeat Domain [2] (Fig. 6A). TCOF1 is involved in the transcription of ribosomal DNA (rDNA) genes, a critical process for the formation and proliferation of NCCs and the subsequent development of craniofacial tissues [16]. Two primary pathogenic mechanisms have been proposed for how TCOF1 mutations lead to TCS: one involves decreased ribosomal RNA (rRNA) production, and the other implicates disruption of telomere stability [17, 18]. Valdez et al*.* [19] demonstrated that reduced TCOF1 expression leads to inadequate rRNA production and aberrant ribosome formation, adversely affecting NCCs development. Conversely, Xin et al*.* [11] reported that TCOF1 deficiency can result in telomere replication anomalies, leading to fragile telomeres and genomic instability, thereby impairing NCCs viability and proliferation, contributing to TCS. In our study, we observed that the mutated TCOF1 failed to fully translocate into the nucleus and exhibited abnormal interactions with Pol II, potentially disrupting DNA transcription and consequently leading to aberrant NCCs development.

In this study, all identified mutations are heterozygous. The concept of haploinsufficiency as a potential pathogenic mechanism is supported by findings from Dixon et al*.* [20], who reported that mice embryos haploinsufficient for Tcof1 exhibited severe cranio-skeletal defects. This suggests that Tcof1 plays a critical role in the formation and proliferation of NCCs, with its deficiency leading to a reduced number of cranial NCCs. Furthermore, during transcription, the CRD and CTD of TCOF1 interact with RNA polymerase I (Pol I), RNA polymerase II (Pol II), Upstream Binding Factor (UBF), and other genes involved in transcription within the nucleus [19, 21] (Fig. 5B). Our study’s findings indicate that the mutant TCOF1, due to its impaired nuclear entry, could not interact normally with Pol II, possibly affecting DNA transcription and leading to abnormal NCCs development.

Approximately 73% of the TCOF1 protein consists of intrinsically disordered regions, which facilitate extensive interactions with other proteins through phase separation. This capability is crucial for executing various physiological and pathological processes within cells [22–26]. The identified mutation induces alterations in the disordered region of TCOF1, potentially resulting in abnormal phase separation and consequent functional impairment. Furthermore, the absence of the C-terminus due to the mutation precludes the recognition of the rRNA promoter and UBF recruitment. Such a disruption may impede the formation of the CUL3^KBTBD8^-NOLC1-TCOF1 complex, a critical platform for ribosome biogenesis, particularly in NCCs [27] (Fig. 5B).

The efficacy of antenatal examination for TCS is currently hindered by the extensive spectrum of identified pathogenic variants and the notable genotype–phenotype mismatch associated with the syndrome. Consequently, there is an urgent need to continue identifying pathogenic mutations in TCS. This will not only enhance the precision of antenatal examinations but also offer more informed guidance in genetic counseling. Furthermore, elucidating the sequence-structure–function relationship of TCOF1, particularly how protein truncation leads to abnormal phase separation, is crucial. Such understanding could significantly refine our knowledge of the etiology of TCS.

## Conclusion

This study presents a comprehensive mutational analysis of 12 Chinese patients clinically diagnosed with Treacher Collins syndrome (TCS), accompanied by an in vitro functional examination of a pathogenic mutation identified in one TCS family. Our findings, in conjunction with previous research, indicate that pathogenic mutations in TCOF1 predominantly occur in two core domains: the Central Repeat Domain (CRD) and the C-terminal Nuclear Localization Sequence (CTD with NLS). Besides the de novo frameshift mutation, TCOF1 c.1559_1571del p.(A521Hfs*71) identified in a Chinese TCS family, other frameshift mutations like p.(N574Efs*29), p.(Q925Pfs*7)/p.(A926Hfs*9), and p.(K1464Rfs*112) may affect TCOF1’s interaction with other transcriptional factors and could impede the development of NCCs. Our study further refines the spectrum of TCOF1 mutations in the Chinese patient population. This study contributes additional clinical evidence and mutational data, enhancing genetic counseling for TCS and serving as a valuable reference for future research endeavors.

## Supplementary Information

Additional file 1: Figure S1. Prediction of Intrinsically Disordered Regions in TCOF1. This figure illustrates the analysis predicting that approximately 73% of the amino acids in TCOF1 are located within intrinsically disordered regions, highlighting the protein's propensity for structural flexibility.

## Data Availability

The datasets used and/or analysed during the current study are available from the corresponding author on reasonable request.
