# Supplementary figures and images for: Deciphering TCOF1 mutations in Chinese Treacher Collins syndrome patients: insights into pathogenesis and transcriptional disruption
Source: Orphanet J Rare Dis. 2025 Feb 7;20:57. doi: 10.1186/s13023-024-03508-z (PMC11806786; doi:10.1186/s13023-024-03508-z)

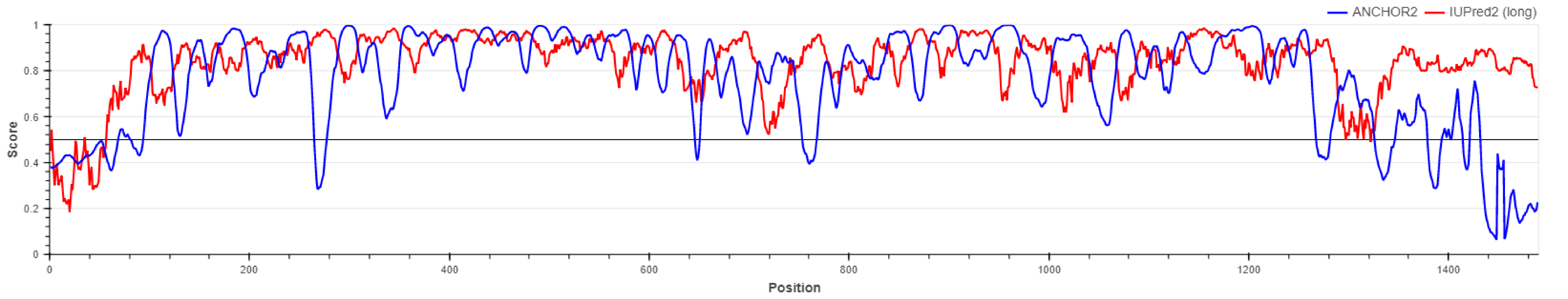

Supplement: Supplementary file 1 — Additional file 1: Figure S1. Prediction of Intrinsically Disordered Regions in TCOF1. This figure illustrates the analysis predicting that approximately 73% of the amino acids in TCOF1 are located within intrinsically disordered regions, highlighting the protein's propensity for structural flexibility. [file 13023_2024_3508_MOESM1_ESM.tif]
